# Unveiling the lamellar structure of the human cornea over its full thickness using polarization-resolved SHG microscopy

**DOI:** 10.1038/s41377-023-01224-0

**Published:** 2023-08-02

**Authors:** Clothilde Raoux, Anatole Chessel, Pierre Mahou, Gaël Latour, Marie-Claire Schanne-Klein

**Affiliations:** 1 Laboratory for Optics and Biosciences, Ecole Polytechnique, CNRS, INSERM, Institut Polytechnique de Paris, 91128 Palaiseau, France; 2 Université Paris-Saclay, 91190 Gif-sur-Yvette, France

**Keywords:** Multiphoton microscopy, Nonlinear optics, Polarization microscopy, Biophotonics

## Abstract

A key property of the human cornea is to maintain its curvature and consequently its refraction capability despite daily changes in intraocular pressure. This is closely related to the multiscale structure of the corneal stroma, which consists of 1–3 µm-thick stacked lamellae made of thin collagen fibrils. Nevertheless, the distribution, size, and orientation of these lamellae along the depth of the cornea are poorly characterized up to now. In this study, we use second harmonic generation (SHG) microscopy to visualize the collagen distribution over the full depth of 10 intact and unstained human corneas (500–600 µm thick). We take advantage of the small coherence length in epi-detection to axially resolve the lamellae while maintaining the corneal physiological curvature. Moreover, as raw epi-detected SHG images are spatially homogenous because of the sub-wavelength size of stromal collagen fibrils, we use a polarimetric approach to measure the collagen orientation in every voxel. After a careful validation of this approach, we show that the collagen lamellae (i) are mostly oriented along the inferior–superior axis in the anterior stroma and along the nasal-temporal axis in the posterior stroma, with a gradual shift in between and (ii) exhibit more disorder in the anterior stroma. These results represent the first quantitative characterization of the lamellar structure of the human cornea continuously along its entire thickness with micrometric resolution. It also shows the unique potential of P-SHG microscopy for imaging of collagen distribution in thick dense tissues.

## Graphical abstract

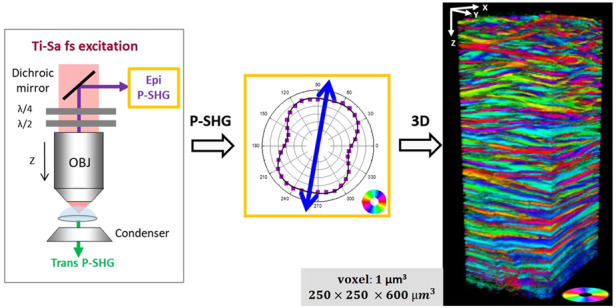

## Introduction

The cornea is the most anterior segment of the eye and is characterized by its transparency and refractive power^1^. Its mechanical properties are also crucial for maintaining the same curvature and the subsequent refraction capability despite daily changes in intraocular pressure and external shocks. All these properties are closely related to the structure of the stroma, the main layer of the cornea^2^. The stroma is around 500 µm thick in the cornea center and consists of 1–3 µm-thick stacked lamellae made of collagen fibrils (around 30 nm in diameter) aligned and regularly packed to ensure cornea transparency^1^. These lamellae are roughly organized parallel to the cornea surface, with different collagen orientations in sequential lamellae. However, the distribution of these lamellae as well as their size and orientation to the cornea surface vary along the depth of the cornea. For instance, in the anterior part of the stroma, lamellae are interwoven and collagen fibrils can run from one lamella to the next one, while lamellae seem to be more regularly stacked in the posterior stroma^3^. Altogether, the anisotropic hierarchical organization of the cornea and its physiological consequences on cornea biomechanics have not been accurately characterized yet because of the limitations of conventional techniques.

Transmission and scanning electron microscopies are highly powerful imaging methods that can resolve the collagen fibrils within the lamellae and show the steep changes of collagen orientation between adjacent lamellae along the corneal depth^4,5^. However, they are two-dimensional imaging techniques with a limited field of view, which is not suitable for a precise characterization of 500 µm-thick tissue around 10 mm in diameter. Optical coherence tomography (OCT) and reflectance confocal microscopy are three-dimensional (3D) imaging techniques that are routinely used in clinical ophthalmology, but they can hardly resolve collagen lamellae and cannot visualize their orientation^2,6^. X-ray scattering has been used successfully to measure the average orientation of collagen lamellae over the full thickness of the cornea^7^. It has shown that collagen lamellae are mainly oriented along the nasal–temporal and inferior–superior axes in the center of the cornea, while an orientation tangential to the circumference predominates in the periphery^7^. Moreover, micro-focus X-ray scattering on thick transverse sections of corneas has shown a difference in the inclination of these lamellae from the center to the periphery and through the depth^8^. However, this technique cannot resolve the in-plane collagen orientation within lamellae through the depth of the stroma and has limited transverse resolution.

Second harmonic generation (SHG) microscopy is nowadays the gold standard technique for in situ visualization of collagen 3D organization in unstained biological tissues^9,10^. This multiphoton modality has already been used in human corneas in combination with two-photon excited fluorescence (2PEF) to visualize keratocytes, which are cells within the stroma^11–21^. However, usual epi-detected SHG images with no polarization resolution are homogeneous, except for low signal lines related to stromal striae that correspond to undulations of lamellae and may have a mechanical role to absorb increases in intraocular pressure and external shocks^22^. On the other hand, trans-detected SHG images exhibit a longer coherence length, which can encompass collagen fibrils with opposite polarities, and thus results in striated patterns oriented along the collagen fibrils. Unfortunately, collagen orientation is not processed efficiently in the numerous voxels showing cross striations. Moreover, trans-detection is not compatible with usual corneal chambers that reproduce physiological conditions^2^. Accordingly, no complete multiscale structure of the human cornea has been reported up to now.

In this study, we have implemented polarization-resolved SHG (P-SHG) microscopy^23–26^ and shown that epi-detection of P-SHG signals is an efficient and precise method to visualize the collagen orientation over the whole depth of human corneas (500–600 µm). We have optimized our P-SHG imaging protocols to get reliable P-SHG data in depth and implemented a data-processing workflow to estimate automatically the mean collagen orientation in every voxel as well as the precision of this measure. Using this approach, we have obtained 3D reconstructions of collagen orientation over the full depth of 10 whole human corneas as well as the lamellae orientation distribution along the depth of the cornea.

## Results

### Collagen orientation maps over the full thickness of the human cornea

**Fig. 1:**
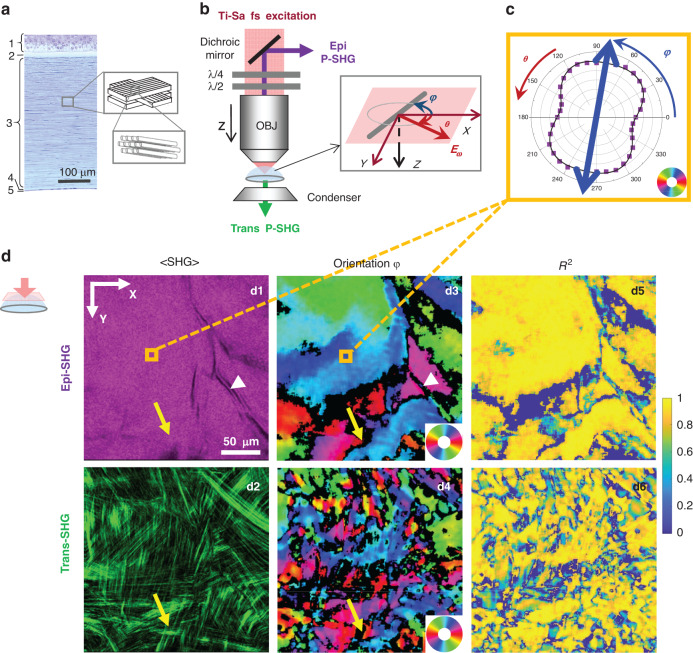
Principle of P-SHG en-face imaging of an unstained human cornea (Cornea no. 1 in Supplementary Table S1). **a** Histology of the cornea and tentative scheme of the stromal structure showing alternating collagen lamellae with perpendicular orientations. 1: epithelium, 2: Bowman’s layer, 3: stroma, 4: Descemet’s membrane, 5: endothelium. **b** Experimental setup showing epi- and trans-detection of SHG images upon excitation at different polarization angles *θ* by use of waveplates. The inset shows the geometry of a stromal collagen fibril and of the excitation electric field within the microscope frame *XYZ*. **c** Polar plot of the epi-detected P-SHG signal in the yellow rectangle in (d1) and (d3) images. **d** Epi-detected (top) and trans-detected (bottom) <SHG> images (d1–d2), orientation images (d3–d4) and *R*^2^ images (d5–d6). The collagen orientation within the imaging plane *XY* is coded as shown in the color wheel. White arrowheads show stromal striae. Yellow arrows show regions encompassing 2 lamellae with different collagen orientations

We measured the collagen orientation in the center of 10 human corneas using P-SHG microscopy. Figure 1a displays a transverse histological section of a human cornea and a tentative scheme of the collagen lamellar structure in the stroma. P-SHG image stacks are recorded over the full thickness of the corneas using en-face geometry (Fig. 1b, c). We obtain three types of information in every pixel: (i) the <SHG> image obtained as the pixel-wise average of the SHG signals over all linear polarizations, which is similar to the usual SHG image acquired with circularly polarized excitation and the same total acquisition duration, (ii) the map of the in-plane orientation *φ* of collagen fibrils and (iii) the map of the coefficient of determination *R*^2^.

**Fig. 2:**
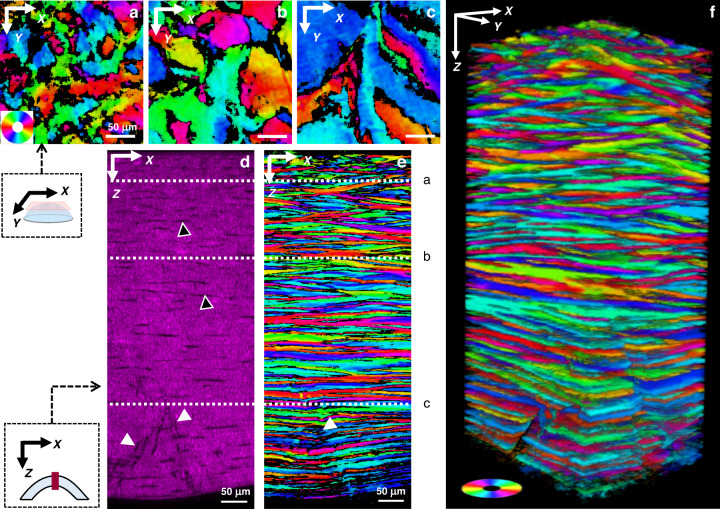
Epi-detected P-SHG en face imaging of an unstained human cornea (Cornea no. 1 in Supplementary Table S1). **a**–**c** En-face orientation images at depths 55 µm (**a**), 210 µm (**b**) and 500 µm (**c**) processed from epi-detected P-SHG stacks. The collagen orientation within the imaging plane *XY* is coded as shown in the color wheel. **d** and **e** Transverse reconstructions of **e** the <SHG> signals and **f** the orientation maps. The dotted white lines indicate the depth of the en-face images (**a**–**c**). **f** 3D reconstruction of the orientation maps processed from the P-SHG image stack. White arrowheads show stromal striae and black arrowheads show keratocytes

Typical <SHG> images using epi- and trans-detections are displayed in Fig. 1d1–d2. They show different features due to different coherence lengths of the SHG process. The larger coherence length in the forward direction enables signal build-up over a larger scale (a few µm) and results in constructive or destructive interferences depending on the relative polarity of fibril domains within this coherence length. The trans-detected <SHG> image accordingly displays striated features that reflect the orientation of collagen fibrils within lamellae^16^. Conversely, the small coherence length in the backward direction results in the signal build-up on a smaller scale (≈100 nm) and almost homogeneous images, except for long areas with no signal. These structures can be attributed either to stromal striae or to keratocytes. Transverse reconstructions of the epi-detected <SHG> images (Figs. 2d and S5a) show that the stromal striae generally run obliquely from the Descemet’s membrane in the posterior stroma (white arrowheads), as already reported^22^. They are also slightly visible in the trans-detected <SHG> images (Fig. S5b). Keratocytes, which lie between lamellae, are oriented parallel to the cornea surface, as clearly observed in transverse reconstructions (Figs. 2d and S5a, black arrowheads).

Collagen in-plane orientation maps obtained from P-SHG data are displayed in Fig. 1d3–d4. They show domains with similar orientations that correspond to lamellae (Fig. 1a). As expected, these orientations obtained by P-SHG are consistent with the orientations of the striations in the trans-detected <SHG> image (Fig. 1d2)^16^. Orientations are not determined reliably in every pixel as shown by the *R*^2^ maps shown in Fig. 1d5–d6. Pixels with low *R*^2^ are displayed as black pixels (*R*^2^ < 0.7) and correspond either to stromal striae (white arrowheads) or keratocytes where the SHG signal is too low or to crossings of lamellae (yellow arrows) as observed in the trans-detected <SHG> image. In the latter case, the focal volume contains fibrils with different orientations, so that the overall cylindrical symmetry does not apply and Eqs. (2) and (3) are not valid anymore, resulting in low *R*^2^.

Figure 1d3–d4 show that the collagen orientation maps obtained from epi- and trans-detected signals are different: domains with similar orientations, which correspond to lamellae, are larger and have smoother boundaries in epi orientation maps, while the trans orientation maps appear somewhat grainy, with more black pixels, as also observed in the *R*^2^ maps (Fig. 1d5–d6). Similarly, transverse reconstructions of epi-detected <SHG> show well-defined lamellae (Figs. 2e and S6a), while those of trans-detected <SHG> show lines with varying thickness and numerous interruptions (Fig. S6b). Here again, this is attributed to the different coherence lengths. Due to its larger coherence length, trans-detected <SHG> probes a larger axial volume that is more prone to contain fibrils of different directions in 2 sequential lamellae. Conversely, the coherence length in epi-detected <SHG> is smaller than the lamella thickness (≈1–3 µm), so it probes fibrils oriented the same way, and this orientation is determined reliably with *R*^2^ > 0.7. Consequently, only epi-detected orientation maps are processed in the following to obtain quantitative information.

Figure 2a–c display orientation maps obtained from epi-detected P-SHG at increasing depths within the stroma. It shows that the size of the lamellae increases from the anterior stroma to the posterior stroma. This increase is regular all along the stroma as shown in the Sup. Movie M1. Figure 2e, f display the transverse and 3D reconstructions of these orientation maps. It clearly shows stacked lamellae with different orientations, depicted as different colors. The angular shift between two sequential lamellae often appears to be close to 90°, but not always so. Moreover, the lamellae in the posterior stroma are parallel to the corneal surface, while the lamellae in the anterior stroma appear to be anchored to the Bowman’s layer with an oblique orientation to the corneal surface. All these observations are in agreement with previous reports^4,8,15^. Most importantly, such 3D maps of collagen in-plane orientation are recorded in 10 human corneas and show consistent results (Fig. S7).

### Validity of collagen orientation measurements in depth

**Fig. 3:**
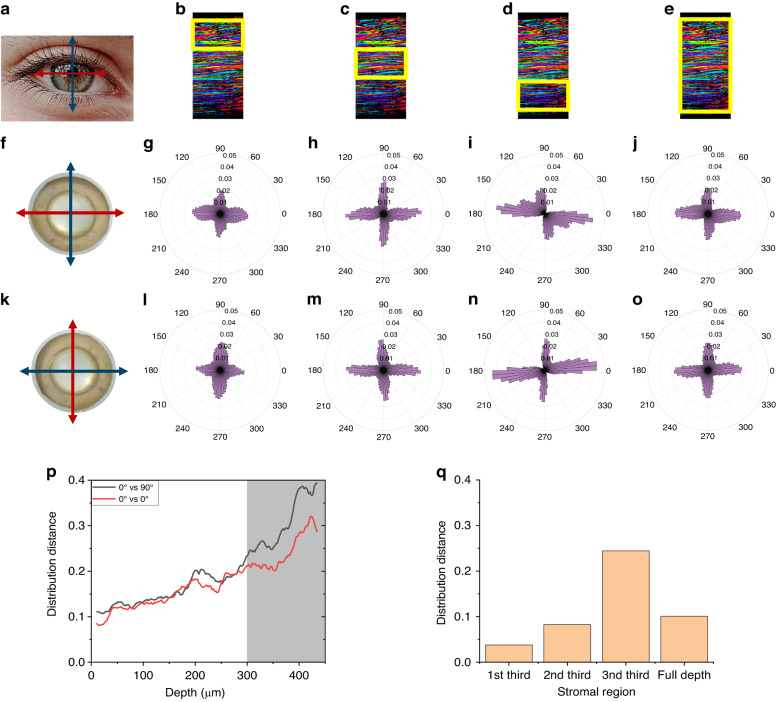
P-SHG orientation distributions for 2 perpendicular orientations of a human cornea (Cornea no. 7 in Supplementary Table S1). **a** Scheme of the nasal-temporal (red double arrow) and inferior–superior (blue double arrow) axes of the human cornea. **b**–**e** Transverse reconstruction of the P-SHG image highlighting in yellow color the regions where the histograms are computed: **b** 1st part, **c** 2nd part, **d** 3rd part, **e** full thickness. **f** Corneal imaging with the nasal-temporal axis along the *X*-axis; **g**–**j** polar plots of collagen orientation distribution obtained with this geometry. **k**–**o** Same for the cornea oriented with the nasal–temporal axis along the *Y*-axis. Histograms are processed using 1° bins and plotted using 5° bins. **p** Distance of the 2 P-SHG orientation distributions obtained for 2 perpendicular (black line) or identical (red line) orientations of the cornea as a function of the depth within the stroma. **q** Distance of the 2 P-SHG orientation distributions obtained for 2 perpendicular orientations of the cornea, processed in different regions of the stroma and over its full thickness. The distances are normalized distances in the range [0,1]

As P-SHG is sensitive to polarization distortions^25^, we have implemented two approaches to verify the validity of these orientation measurements and assess their precision. The first approach applies to the orientation distributions in the field of view obtained at every depth or in specific axial regions. It compares the orientation distributions processed from a series of P-SHG acquisitions for different orientations of every cornea: the nasal-temporal corneal axis (Fig. 3a) is first aligned along *X*-axis (P-SHG-1), then along *Y*-axis (rotation by 90°, P-SHG-2) and finally again along *X*-axis (P-SHG-3). Figure 3 displays polar plots of the orientation distributions processed in the first, second, and third parts of the stroma, considered here as the anterior, middle, and posterior stroma, as well as over the full thickness of the stroma (Fig. 3b–e), for the two perpendicular orientations (P-SHG-1 and P-SHG-2) of the same cornea (Fig. 3f–o). Polar plots in the anterior stroma rotate by 90° when the cornea is rotated by 90° as expected. The same behavior is observed in the middle stroma, although less clearly. However, this behavior is not observed in the posterior stroma, which questions the reliability of P-SHG data in this region. We have verified that this artifact is not specific to the posterior stroma, but appears in depth whatever the orientation of the cornea, that is in the anterior (resp. posterior) stroma when the cornea is oriented with the posterior (resp. anterior) stroma facing the objective. This artifact is thus attributed to slight distortions of the excitation polarization in-depth, most probably due to a combination of slight defects: (i) not perfect centering of the cornea apex and residual field curvature of the objective^27^; (ii) slight optical heterogeneities of the cornea, inducing mild depolarization and birefringence.

We have quantified this artifact as a function of the imaging depth within the cornea by calculating the normalized distance (Eq. (6), Fig. S4) between the orientation distributions processed for the different cornea orientations (Fig. 3p, q). The two distributions are first angularly registered so that the distance is expected to be 0 for perfect measurements. This distance is plotted at every depth in Fig. 3p: in black color for P-SHG-1 and P-SHG-2, ie for 2 perpendicular orientations of the cornea, and in red color for P-SHG-1 and P-SHG-3, ie for 2 identical orientations. We observe that P-SHG measurements are not perfectly identical for the same orientation of the cornea. This nonzero control distance is attributed to the imperfect axial repositioning of the cornea after +90° and −90° manual rotations of the cornea holder, considering the high sensitivity of SHG signals to the exact location of every pixel due to its coherent nature. The distance between the P-SHG measurements with perpendicular orientations of the cornea is identical to this control distance in the two first thirds of the stroma, which validates the P-SHG measurements in these regions. However, the distance increases in the last third, in a higher manner than for the control distance, which indicates the presence of an artifact. This artifact is better quantified by computing the distribution distance over the three-thirds of the stroma (Fig. 3q). The distance is <0.1 in the two first thirds, while it increases to 0.25 in the posterior region. Note that measurement over the full depth of the stroma cannot evidence these artifacts because of the averaging process (Fig. 3q).

**Fig. 4:**
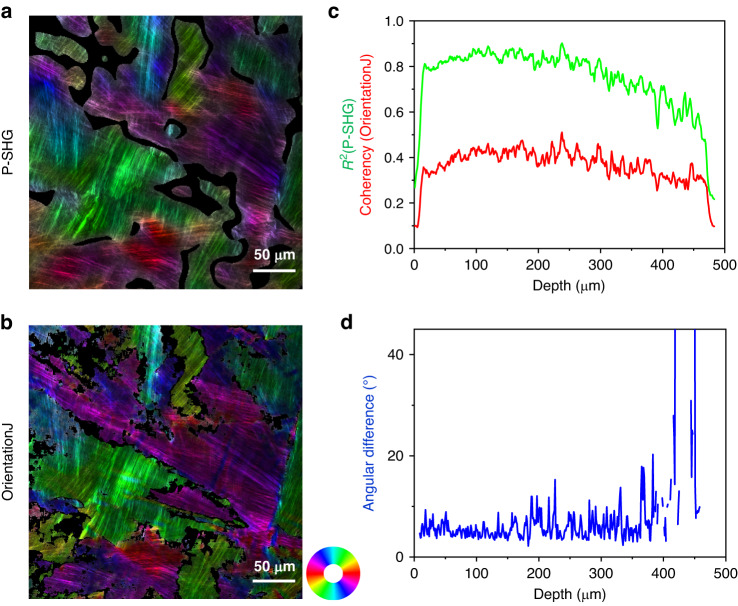
Precision assessment of orientation maps obtained from P-SHG. **a** Orientation map obtained from epi-detected P-SHG imaging using HSV color map superimposed on the trans-detected <SHG> image; H codes the orientation as shown in the color wheel, *S* = *R*^2^, *V* is the trans-detected <SHG> signal. Black pixels correspond to *R*^2^ < 0.7. **b** Orientation map obtained from image processing of the trans-detected <SHG> image that is acquired simultaneously, using a similar HSV color map, with *S* being the coherency. Black pixels correspond to Coherency < 0.2. **c** Depth profiles of the *R*^2^ and coherency parameters without any filtering. **d** Mean angular difference of the 2 orientation maps as a function of the depth within the stroma, after filtering out the pixels with *R*^2^ < 0.7 or coherency < 0.7. The mean angular difference in the first 2/3 region of the stroma is 5.6°

The second validation approach applies to every voxel of the orientation maps. The ground truth is provided by the simultaneous acquisition of the trans-detected <SHG> images that show striations parallel to the collagen fibrils. We have thus compared pixel-by-pixel the orientation maps obtained from epi-detected P-SHG images to the ones obtained by image processing of trans-detected <SHG> images using OrientationJ^28^. Figure 4a, b display the two resulting orientation maps superimposed on the trans-detected <SHG> image. They look similar but not perfectly identical. The main issue is that image processing is unable to extract valid orientations from regions with crossing striations, as shown by the low value of the coherency parameter that assesses OrientationJ precision (Fig. 4c). Indeed, due to the large coherence length in the trans-direction, SHG signals are often obtained from two superimposed lamellae with different collagen orientations. In contrast, epi-detected SHG signals advantageously exhibit a low coherence length and consequently most often build up in a unique lamella. Accordingly, the *R*^2^ parameter is quite high all along the thickness of the cornea (Fig. 4c). We have therefore filtered out the orientations obtained by the two methods using an identical threshold for R^2^ and the coherency (<0.7) and processed the mean angular difference between the two maps at every depth using Eq. (7). This difference is typically 6° in the two first thirds of the stroma and increases deeper in the stroma (Fig. 4d). P-SHG orientation maps are thus reliable up to approximately 2/3 of the cornea thickness and their effective precision is around 4°, considering that the measure uncertainty is similar for OrientationJ and P-SHG and using error propagation. This effective precision is much larger than the one usually claimed for P-SHG data that is assessed from theoretical analysis or provided by fitting or FFT codes^29,30^. It indeed takes into account all experimental uncertainty sources in thick tissues, including the tissue optical heterogeneity that induces wavefront deformation, focal volume deterioration, and ellipticity increase even at moderate depth.

In conclusion, both assessment approaches indicate that epi-detected P-SHG provides reliable orientation measurements with 4° precision in the two first thirds of the corneal stroma (up to around 350 µm). Nevertheless, Fig. 3i, n show that the deformation of the orientation distributions in the last third of the stroma is similar for two perpendicular orientations of the cornea. Averaging of these two distributions can therefore efficiently compensate for the two opposite deformations. We have therefore implemented the following protocol: (i) sequential acquisitions of two P-SHG image stacks in the same cornea oriented with the naso-temporal axis along the *X* and *Y* directions; (ii) numerical rotation by 90° of the orientation distributions extracted from the second P-SHG stack and averaging with the distributions extracted from the first P-SHG stack (Movie M2). This procedure provides reliable distributions of the collagen orientation over the full cornea thickness. It is however not directly applicable to the image stacks, even after spatial registration, because SHG, as a coherent process, is highly sensitive to the exact voxel position (within a few 10 s of nm) that is not perfectly reproducible in sequential z-stacks acquisitions, as already noted^31^. The last third of the 3D orientation reconstruction (Fig. 2e, f) must therefore be considered as qualitative images showing alternating lamellae with perpendicular orientations, and only the orientation distribution averaged over the two perpendicular P-SHG stacks is quantitative data.

### Quantitative analysis of lamellae orientation distribution

**Fig. 5:**
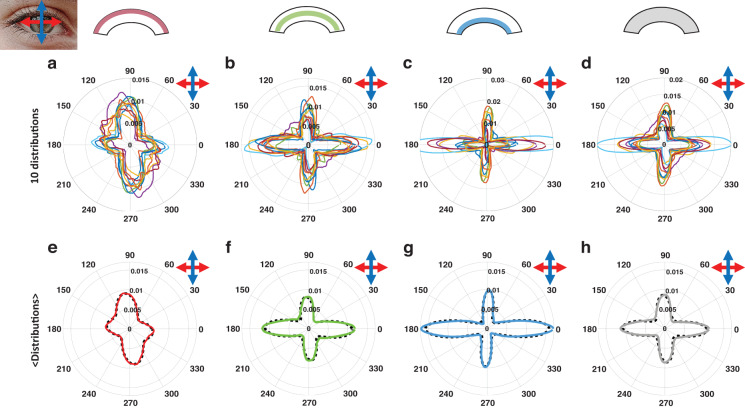
Collagen orientation distributions in 10 human corneas. **a**–**d** Distributions in each cornea and **e**–**h** averaged distribution in the (**a**, **e**) first, (**b**, **f**) second, and (**c**, **g**) third parts of the stroma and (**d**, **h**) in the full stroma. The solid lines correspond to experimental data and the dotted lines to fits of the averaged distributions using Eq. (4). The distribution scales are the same in (**e**–**h**), while they are adjusted in (**a**–**d**) to better visualize the different lines. All polar distributions are plotted with the nasal-temporal axis along the *x*-axis

Following this approach based on two sequential P-SHG acquisitions for two perpendicular orientations of the cornea, we obtain collagen orientation distributions that represent *in fine* the orientation distribution of stromal lamellae. Figure 5a–d shows polar plots of these distributions for each of the 10 human corneas under study. They are computed either in each of the three thirds of the cornea thickness or in the whole cornea. They are highly reproducible in the 10 corneas and consistently display two perpendicular peaks along the nasal–temporal and the inferior–superior directions. These peaks are more prominent in the posterior stroma (Fig. 5c) than in the anterior one (Fig. 5a), and the predominant orientation shifts from the inferior-superior axis in the anterior stroma (Fig. 5a) to the nasal–temporal axis in the posterior stroma (Fig. 5c).

In order to quantify these features, the orientation distributions of all corneas are averaged and the averaged distributions are fitted using Eq. (4) (Fig. 5e–h). The fitting parameters are displayed in Table 1, including the fit *R*^2^. This parameter is close to 1, which indicates that the orientation distributions are well described by the sum of two Van Mises distributions and an isotropic component. The angular shift Δ*μ* between the two peaks is 86° (resp. 83°, 87°) for the orientation distribution over the whole cornea thickness (resp. in the 2nd and 3rd parts), which is very close to 90°. This is fully consistent with the usual description of the human cornea as a stacking of collagen lamellae with two main perpendicular orientations along the nasal-temporal and inferior–superior axes. However, our results show that this usual description must be reconsidered in the anterior stroma: the two peaks are not well-defined in all corneas, so the average distribution show wide peaks (small *κ* parameters) that are not exactly perpendicular (Δ*μ* = 74°). Moreover, our data highlight variations in the lamellae distribution along all the stroma. First, the peaks become thinner and thinner from the anterior to the posterior stroma, as quantified by the continuous increase in the *κ* parameters. It means that the lamellae are better aligned along two perpendicular axes in the posterior stroma than in the middle one and even less in the anterior one. Second, the main orientation of the lamellae shifts gradually from the inferior–superior axis in the anterior stroma to the nasal–temporal axis in the posterior stroma, as quantified by the increase in the *c*_NT_ parameter and the concomitant decrease in the *c*_IS_ parameter. This feature is not visible in the full-thickness distribution where the variations are averaged, which results in well-balanced peaks with a similar percentage of lamellae along the two perpendicular axes. The same averaging applies to all techniques with no 3D resolution, notably to X-rays scattering data that are recorded on the full thickness of the cornea. Accordingly, the orientation distribution obtained by P-SHG on the full thickness of the cornea is in good agreement with the one obtained by X-ray scattering by Meek et al^7,32^. Note however that the isotropic component is quite strong in X-ray data, while it is minor in P-SHG data, as already reported^33^. This effect is nevertheless difficult to quantify because the isotropic coefficient in Eq. (4) is partially dependent on the *κ* parameters as wider peaks may decrease the isotropic part.

**Table 1:** Fitting parameters of orientation distributions in human cornea

Parameter	Anterior part	Middle part	Posterior part	Full thickness
Δ*μ*	74°	83°	87°	86°
*c*_iso_	36%	47%	41%	54%
*c*_NT_	23%	32%	39%	26%
*κ*_NT_	1.6	3.9	6.4	5.2
*c*_IS_	41%	21%	20%	20%
*κ*_IS_	1.8	3.7	7.4	4.1
*R*^2^	0.99	0.97	0.99	0.97

## Discussion

In this study, we have implemented advanced P-SHG imaging protocol and processing to measure the collagen orientation over the full thickness of 10 entire human corneas with micrometric resolution. Orientation measurement by P-SHG is nevertheless an indirect and complex method that deserves careful validation. Our study shows that reliable orientation measurements rely on the following key elements, besides the use of an objective lens with high numerical aperture and of highly-sensitive detectors: (i) use a custom-designed cornea holder that maintains physiological conditions, mainly the cornea curvature, and minimize polarization artifacts due to tissue deformation; (ii) carefully align the microscope setup and optimize the linearity of the excitation polarization, in order to maximize the contrast of the polarimetric diagram in every voxel; (iii) take advantage of epi-detection to record SHG images with low coherence length and maximize the number of voxels encompassing unique lamellae, with well-defined collagen orientation, rather than two lamellae with two different orientations; (iv) implement an automatic image-processing workflow to calculate the collagen orientation in every voxel from the P-SHG images and eliminate unreliable data based on *R*^2^ filtering.

These key points are all related to the sensitivity of polarimetric measurements to any optical defect. Accordingly, P-SHG microscopy has been reported to be prone to many artifacts, especially in depth^30,34,35^. As the cornea is transparent and exhibits globally a very low birefringence, we expect negligible issues due to scattering or birefringence, but the polarimetric diagrams may be slightly distorted in-depth due to slight misalignment or tissue heterogeneities. Most importantly, the focal volume can encompass two sequential lamellae composed of collagen with different orientations, which invalidates the cylindrical symmetry hypothesis used to analyze P-SHG data^36^. This effect is much lower in epi-detection, because of the much smaller coherence length, but it is still present in a few voxels. We have therefore filtered out the voxels where the polarimetric data do not fit the theoretical Eq. (3) by calculating a coefficient of determination *R*^2^ and eliminating voxels with *R*^2^ < 0.7 (Fig. S3). We may alternatively detect a violation of cylindrical symmetry by fitting P-SHG data with a theoretical equation based on trigonal symmetry^37^ or by looking for discrepancies between *φ*_2_ and *φ*_4_ (see Supplementary Material)^38,39^. Application of an *R*^2^ threshold is anyway easy to implement and efficient to eliminate unreliable P-SHG data^40^.

Beyond this careful filtering of the P-SHG data, we have also validated these orientation measurements by two complementary methods. This is mandatory because FFT processing of P-SHG data always provides an orientation, whatever the matching of the polarimetric response to the theory. First, we have verified that the orientation distributions obtained by P-SHG rotate when the cornea is physically rotated under the objective lens, which advantageously requires no ground truth. Second, we have compared in every voxel the orientations obtained by epi-detected P-SHG and by processing the trans-detected <SHG> images using OrientationJ, considered as the ground truth. Both methods are consistent in indicating that epi-detected P-SHG measurements of collagen orientation in the cornea are valid until approximately 350 µm with about 4° angular precision. Nevertheless, collagen orientation distributions can be measured deeper in a reliable way by averaging a series of measurements performed with two perpendicular orientations of the cornea. This practical protocol provides quantitative measurements of the lamellae orientation distribution along the full thickness of the cornea which is up to 600 µm.

We have used this optimized protocol to visualize and quantify the lamellar structure of 10 entire human corneas. We have used a large field of view encompassing few lamellae (250 µm) and a small voxel size enabling lamellar resolution in the axial direction (1 µm axial step) in order to better visualize the lamellar domains than in previous reports^16,33^. This is not possible using image processing of trans-detected <SHG> data because of the larger coherence length, as already noted^18,19,41^. Alternatively, the use of polarization-resolved third harmonic generation (THG) microscopy^42,43^ may be explored, but certainly at the expense of smaller signals that would require longer acquisition times, and only using trans-detection.

3D reconstructions of P-SHG data thus show in a unique way the stacking of collagen lamellae with different orientations all along the thickness of the cornea. It confirms that lamellar domains are larger and better-aligned parallel to the corneal surface in the posterior stroma than in the anterior one^4,8,15^. Compared to previous studies, our data further show the evolution of the lamellae size and orientation in 3D all along the stroma in the very same cornea. As a perspective, these lamellar domains may be individually segmented and quantified. It would nevertheless require to implement new approaches as established segmentation methods do not apply to orientation data that are circular data (0° = 360°), while our quantitative measures of the collagen orientation distributions are already highly informative. They consistently show in all the corneas under study that the lamellae are mostly aligned along the nasal-temporal and the inferior-superior directions, in agreement with the literature^7,32^. They further provide new quantitative information about the structural variations along the corneal depth that cannot be obtained by other techniques: first, the lamellae better align along two perpendicular axes in the posterior stroma than in the anterior one, which shows a more disordered distribution. Second, the collagen lamellae are mostly oriented along the inferior–superior axis in the anterior stroma and along the nasal–temporal axis in the posterior stroma, with a gradual shift in between.

These structural data are crucial for the understanding of the cornea function. The refraction capability of the cornea is indeed directly linked to its curvature, which must therefore be kept constant despite daily variations in the intraocular pressure. This is guaranteed by highly specific mechanical properties that are closely connected to the lamellar structure of the stroma^1,44^. Cornea mechanical properties are routinely estimated using air-puff measurements in vivo, which does not provide extensive mechanical data, and more complete ex vivo measurements are still challenging^33,45,46^. Numerical models are thus mandatory for the prediction of the cornea mechanical properties and refraction capability, but only recent studies have taken into account the lamellar structure of the cornea, still in a simplified manner because of the lack of accurate orientation data^47,48^. This may be overcome by using our depth-resolved lamellae orientation distribution obtained from P-SHG, whereas such depth-resolved information with appropriate resolution and accuracy cannot be obtained using more conventional techniques such as OCT, X-ray scattering, or electron microscopy. This may then enable improved numerical simulation of the mechanical behavior of healthy corneas as well as visually impaired ones. The most direct application would be to provide a better patient-specific prediction of stromal remodeling after refractive surgery and consequently improve the correction accuracy of this surgery. P-SHG may also be used to measure the lamellar structure of keratoconic corneas. Keratoconus is a corneal pathology of increasing prevalence that is characterized by gradual corneal thinning and in the most advanced cases, by the formation of a cone that severely impairs vision. Keratoconus is also characterized by poor mechanical properties (low stiffness), which are associated with a degradation of the stroma structure^18,19,21,22,41,45^. However, no extensive full-depth characterization of the lamellar structure of keratoconic cornea has been reported up to now. P-SHG imaging of keratoconic corneal buttons may bridge this gap and provide crucial data enabling numerical simulations of the mechanical properties of keratoconic cornea^48^. It would be crucial for the understanding of stromal remodeling during keratoconus and it may possibly improve its early diagnosis.

In conclusion, we have taken advantage of the small coherence length in epi-detected SHG microscopy and of P-SHG sensitivity to collagen orientation in order to map the lamellar structure of human corneas with micrometric resolution. We have carefully assessed the precision of our orientation measurements and obtained 3D reconstructions in the center of 10 entire corneas over their full thickness. Quantitative analyses of these data show in a unique way the evolution of the lamellar structure along the depth of the cornea. A key advantage of our method is to use epi-detection and consequently to be compatible with any corneal chamber or with in vivo imaging. It could therefore be used to record stromal remodeling during inflation assays in ex vivo corneas, in order to measure advanced mechanical properties of cornea. Our approach is readily applicable to any tissue and shows the unique potential of epi-detected P-SHG microscopy for imaging thick collagen-rich tissues.

## Materials and methods

### Human cornea samples

This study was carried out according to the tenets of the Declaration of Helsinki and followed international ethical requirements for human tissues. Handling of human corneas was declared to the French administration (CODECOH agreement DC-2018-3300). Ten human corneas were obtained from the French Eye Bank (Banque Française des Yeux, BFY), which were unsuitable for transplantation and authorized for scientific use by the donor family. These corneas were preserved in an appropriate culture medium (Stemalpha 2, StemAlpha or Cornea Max, Eurobio) in an incubator set at 31°. They were then placed for 48 h in Stemalpha 3 without phenol red to induce deswelling and elimination of the phenol red present in the previous culture medium, prior to fixation in 4% paraformaldehyde (PFA) for 24 h at 4 °C. Fixed corneas were stored in 1% PFA at 4 °C until imaging. Physiological data, storage durations, and thicknesses of all corneas are given in Supplementary Table S1.

A specific cornea holder was designed for P-SHG imaging in order to comply with two important requirements: (i) maintain the physiological curvature of the cornea as in usual corneal chambers since we observed polarization artifacts due to diattenuation in pieces of flattened corneas stuck between two coverslips during preliminary P-SHG experiments; (ii) enable trans-detection of SHG signals for validation purpose of epi-detected orientation maps (see below), which is not possible in usual corneal chambers. We used a small plano-convex lens with the same diameter and radius of curvature as the human cornea (LA1576, Thorlabs). The cornea was placed on this lens, in turn, placed on a coverslip, and the sclera was clamped with a plastic ring maintained by a metallic screwed holder (Fig. S1). Ophthalmic gel (Lacrigel, Europhta) was added between the lens and the cornea and between the microscope objective and the cornea in order to avoid cornea dehydration and to enable optical contact. The cornea was imaged in the apex region to ensure that the corneal surface was parallel to the imaging plane and to minimize spherical aberrations. The nasal–temporal and superior–inferior physiological axes of the corneal button were identified and oriented along the *X* or *Y* axis in the imaging plane.

### Polarization-resolved SHG microscopy

SHG images were recorded by use of a custom-built upright laser scanning multiphoton microscope as previously described^21^ (Fig. S2). Excitation was provided by a femtosecond Titanium-Sapphire laser (Mai-Tai, Spectra-Physics) tuned at 860 nm and scanned in the *XY* directions using galvanometric mirrors. A high numerical aperture (NA) objective lens with water immersion (×25, NA 1.05, XLPLN-MP, Olympus) was used with nominal resolutions (FWHM) of 0.35 µm (lateral) × 1.2 µm (axial) and a field of view of 540 × 540 µm^2^. As the cornea refractive index (≈1.37) is different from the one of water, the objective collar was adjusted before each acquisition in order to minimize spherical aberrations and avoid low contrast in depth. It was set to the position that maximized the SHG signal at half the depth of every cornea (typically 250 µm) at the expense of a slightly degraded resolution at the top and bottom of the sample, which was measured as 0.8 µm (lateral) × 2.6 µm (axial). SHG signals were detected in the epi- and trans-directions by means of photon-counting photomultiplier tubes (P25PC, Electron tubes) and suitable spectral filters to reject the excitation beam and select the second harmonic signal at 430 nm (FF01-680SP, FF01-720SP, FF01-427/10 Semrock). Excitation power was linearly increased along the depth of the cornea to compensate for signal decrease, starting from 40 to 55 mW at the epithelium side and reaching 60–100 mW when imaging the endothelium side.

P-SHG imaging was performed by recording a series of SHG images excited by linearly polarized incident electric fields with different orientations (Fig. 1b). The orientation of the incident field polarization was controlled by an achromatic half wave-plate in a motorized rotating mount inserted at the back aperture of the objective (F102978, 30 mm diameter, Fichou). The linearity of the polarization was optimized by an achromatic quarter wave-plate (F102977, 30 mm diameter, Fichou) at the same place. The ellipticity, defined as the ratio of the minimal to maximal electric fields, was smaller than 4% for every polarization in a reduced field of view of 250 × 250 µm^2^. Series of 18 images were acquired every 10° between 0° and 170° using 1 µm pixel size and 5 µs pixel dwell time. Stacks of such P-SHG images were acquired along the full depth of the corneas with 1 µm axial steps, resulting in 2–3 h acquisition per cornea.

### Theoretical analysis of P-SHG images

The SHG response of collagen is described by a second-order nonlinear susceptibility tensor *χ*^(2)^, which links the nonlinear polarization *P*^2*ω*^ to the excitation electric field at fundamental frequency *E*^*ω*^^49^:

1 $${P}_{i}^{2\omega }=\epsilon_0\sum _{j,k}{\chi }_{{ijk}}^{(2)}{E}_{j}^{\omega }{E}_{k}^{\omega }$$

where *i, j, k* stand for *x, y, z*. Considering that the collagen fibrils exhibit a cylindrical symmetry and that the Kleinman symmetry is valid, there are only 2 independent components in the fibril frame *(xyz)*: *χ*_*xxx*_ and ${\chi }_{{xyy}}={\chi }_{{xzz}}={\chi }_{{yxy}}={\chi }_{{yyx}}={\chi }_{{zxz}}={\chi }_{{zzx}}$ where *x* is the fibril axis. The P-SHG signal intensity from a collagen fibril oriented at an angle φ to the *X*-axis in the image plane *XY* then depends on the orientation angle *θ* to the *X*-axis of the linearly polarized excitation field (Fig. 1c)^50^:

2 $${I}_{{{\rm {SHG}}}}(\theta )=K\left[{\left|{\chi }_{{XXX}}{\rm{cos} }^{2}\left(\theta -\varphi \right)+{\chi }_{{XYY}}{\rm{sin} }^{2}\left(\theta -\varphi \right)\right|}^{2}+{\left|{\chi }_{{XYY}}{\rm{sin }}\left(2\left(\theta -\varphi \right)\right)\right|}^{2}\right]$$

The susceptibility components *χ*_*IJK*_ in the microscope frame are equal to the ones *χ*_*ijk*_ in the fibril frame for fibrils lying in the imaging plane, while they also depend on the out-of-plane angle for fibrils lying out of the imaging plane (see Supplementary material). The parameter *K* includes experimental geometrical parameters and the square of the excitation laser intensity.

Equation (2) can be written as a function of its Fourier components *a*_0_, *a*_2,_ and *a*_4_ which are linear combinations of the susceptibility components of the collagen fibrils^23^:

3 $${I}_{{{\rm {SHG}}}}(\theta )={a}_{0}+{a}_{2}\cos \left(2\left(\theta -\varphi \right)\right)+{a}_{4}\cos \left(4\left(\theta -\varphi \right)\right)$$

We thus use an FFT algorithm to process our data and determine the orientation *φ* in every pixel (see Supplementary material). The theoretical angular precision is about 1° after the application of a 6 × 6 averaging filter to increase the signal-to-noise ratio^29,30^. Our data-processing workflow also provides^21^: (i) the SHG signal averaged over all linear polarizations: $\left\langle {{\rm {SHG}}}\right\rangle {=a}_{0}$; (ii) a coefficient of determination *R*^2^ that compares the experimental data and the curve obtained from the FFT parameters in every pixel, in order to validate the orientation measure (Fig. S3). Angles determined with *R*^2^ < 0.7 are eliminated in the quantitative analyses and appear as black pixels. The orientation maps are displayed using an HSV Look-Up-Table: the Hue (*H*) indicates the collagen orientation *φ* and the brightness (*V*) the *R*^2^ parameter.

### Numerical analysis of orientation distributions

Orientation maps obtained from P-SHG data provide the distribution of the collagen orientation in the field of view. These distributions are computed using 1° angular bins either at every depth or in specific regions of the stroma by merging the orientation data over a specific range of depth. Notably, distributions are computed over the full stromal thickness or by dividing the stromal thickness in 3 thirds that correspond approximately to anterior, middle, and posterior stromal regions. The resulting distributions are normalized to the total number of valid pixels and depicted as polar plots. Note that P-SHG provides only the orientation *φ* in the range [0, *π*], not the polarity and that polar plots are therefore extended to *φ* + *π*.

These orientation distributions *p*(*φ*) are finally fitted by the sum of two Van Mises distributions^48^:

4 $$p\left(\varphi \right)=\frac{{c}_{{{\rm {iso}}}}}{\pi }+{c}_{{{\rm {IS}}}}M\left(\varphi |{\mu }_{{{\rm {IS}}}},{\kappa }_{{{\rm {IS}}}}\right)+{c}_{{{\rm {NT}}}}M\left(\varphi |{\mu }_{{{\rm {NT}}}},{\kappa }_{{{\rm {NT}}}}\right)$$

with

5 $$M\left(\varphi |{{\mu }},{{\kappa }}\right)=\frac{{e}^{\kappa \cos \left(2\left(\varphi -\mu \right)\right)}}{{C}(\kappa )}$$

where *C*(*κ*) is a normalization constant so that ${\int }_{0}^{\pi }M\left(\varphi |{{\mu }},{{\kappa }}\right){\rm {d}}\varphi =1$. *μ* and 1/*κ* are analog to the average and the variance of a normal distribution. The angles are doubled in the exponential because *φ* covers only half the full range of directions [0, *π*]. This fitting is applied to the average of the 2 distributions obtained for 2 perpendicular orientations of the cornea, after angular registration, as explained below.

### Computation of distribution distances for 2 orientations of the cornea

In order to verify the validity of these orientation distributions, a series of P-SHG image stacks are acquired and processed for different orientations of every cornea. The orientation distributions, but the first one, are initially angularly shifted to correspond to the same cornea orientation as in the first acquisition. The registration angle is calculated by minimizing the distance of the distributions in the anterior region. This distance between two distributions is calculated as the summation of the absolute difference between the distribution values at each angle (Fig. S4). After registration over the anterior region, it is processed at every depth using a sliding average over 40 µm:

6 $$D\left({Z}_{a},{Z}_{b}\right)={\left\langle \sum _{\varphi }\left|{h}_{a}\left(\varphi ,{Z}_{a}+i\right)-{h}_{b}\left(\varphi ,{Z}_{b}+i\right)\right|\right\rangle }_{i\in \left[-20,+20\right]}$$

Here ${h}_{i}\left(\varphi ,z\right)$ is the orientation distribution at depth *z* for orientation *O*_*i*_ of the cornea. The minimum $D\left({Z}_{a}\right)$ of $D\left({Z}_{a},{Z}_{b}\right)$ then provides the distance of the 2 orientation distributions at every depth *Z*_*a*_. It is normalized to the maximum distance between 2 normalized distributions (i.e. to 2). This distance is also calculated on the orientation distributions computed over the 3 thirds of the stroma and over its full thickness.

### Analysis of trans-detected <SHG> images using OrientationJ

As trans-detected <SHG> images show striations reflecting the orientation of the collagen fibrils, we also determine the orientation distribution by image processing of $\left\langle {SHG}\right\rangle$. We use the ImageJ plug-in OrientationJ, which is based on the local evaluation of the gradient structure tensor^28^. We use a window size of 6 pixels and filter out the pixels where the coherency is smaller than 0.7. We then compare the orientation maps obtained by the two methods in every image of the *z*-stack by computing the average of their pixel-by-pixel absolute difference:

7 $$D\left(Z\right)=\frac{1}{N(Z)}\sum _{x,y}\left|{\varphi }_{P-{{\rm {SHG}}}}\left(X,Y,Z\right)-{\varphi }_{{{\rm {Orient}}J}}\left(X,Y,Z\right)\right|$$

where $N(Z)$ is the number of valid pixels at depth *Z*.

## Supplementary information

Supplementary Information

Movie 1. P-SHG imaging of a human cornea (Cornea n°1 in table S1) over its full thickness

Movie 2. Protocol to obtain reliable orientation distribution of collagen in the posterior stroma

## Data Availability

Raw P-SHG data and processing code in Matlab are available in a Zenodo repository: 10.5281/zenodo.7994819.
