# Supplementary material for: Unveiling the lamellar structure of the human cornea over its full thickness using polarization-resolved SHG microscopy
Source: Light Sci Appl. 2023 Aug 2;12:190. doi: 10.1038/s41377-023-01224-0 (PMC10394036; doi:10.1038/s41377-023-01224-0)
Supplement: Supplementary file 1 — Supplementary Information [file 41377_2023_1224_MOESM1_ESM.pdf]

Supplementary information

for

Unveiling the lamellar structure of the human cornea over its full thickness using polarization-resolved SHG microscopy

CLOTHILDE RAOUX,[1] ANATOLE CHESSEL,[1] PIERRE MAHOU,[1] GAËL LATOUR,[1,2] AND MARIE-CLAIRE SCHANNE-KLEIN[1*]

[1] Laboratory for Optics and Biosciences, Ecole polytechnique, CNRS, INSERM, Institut Polytechnique de Paris, 91128 Palaiseau, France

[2] Université Paris-Saclay, 91190 Gif-sur-Yvette, France

* marie-claire.schanne-klein@polytechnique.edu

1. Supplementary Methods

1.1 Theoretical background of P-SHG

We derive here the polarization-resolved SHG signal from fibrillar collagen in the theoretical framework of tensorial nonlinear optics [1]. SHG process is described by a second-order nonlinear susceptibility tensor $\chi^{(2)}$ that induces a nonlinear polarization $\vec{P}^{(2)}(\vec{r},t)$ at 2ω:

$$P_i^{(2)}(\vec{r}) = \epsilon_0 \sum_{j,k} \chi_{ijk}^{(2)} E_j(\vec{r}) E_k(\vec{r}) \quad \text{(S1)}$$

Here *i,j,k* refer to the Cartesian components of the fields and $\vec{E}(\vec{r},t) = \vec{E}(\vec{r})\, e^{-i\omega t} + cc$ is the fundamental field. We assume as in previous reports that the susceptibility tensor $\chi^{(2)}$exhibits a cylindrical symmetry in the fibril frame (xyz), where x the fibril direction, and that the Kleinman symmetry applies [2,3]. While more complex tensors may be used, these assumptions have been shown to well reproduce P-SHG experimental data, while they limit the number of independent tensor components to only 2: $\chi_{xxx}^{(2)}$ and $\chi_{xyy}^{(2)} = \chi_{xzz}^{(2)} = \chi_{yxy}^{(2)} = \chi_{zxz}^{(2)} = \chi_{yyx}^{(2)} = \chi_{zxx}^{(2)}$. We set $\rho_{fib} = \frac{\chi_{xxx}^{(2)}}{\chi_{xzz}^{(2)}}$, called the fibril anisotropy parameter, which has been shown to provide the orientation δ of the peptide bonds to the triple helical axis: $\rho_{fib} = \frac{2}{\tan^2\delta}$ [4-8].

Let's consider a linearly-polarized fundamental laser beam at angle θ to the axis X in the microscope frame (X,Y,Z):

$$\vec{E}(\vec{r}) = E_0 e^{ikZ} \begin{pmatrix} \cos\theta \\ \sin\theta \\ 0 \end{pmatrix}_{(X,Y,Z)} \quad \text{(S2)}$$

Eq. (2) is written in the plane wave approximation and neglects the axial electric field component along the microscope axis Z due to strong focusing. We have indeed shown previously that strong focusing does not affect the determination of the in-plane orientation φ of collagen fibrils [9]. It only affects the contrast of the polarimetric diagram and impedes accurate measurements of the ratio of the two susceptibility components in the microscope frame $\rho = \chi_{XXX}/\chi_{XYY}$, which is not used in this study.

The SHG signal intensity is then calculated as:

$$I_{SHG} \propto \left(\left| \sum_{J,K} \chi^{(2)}_{XJK} \, E^J_\omega E^K_\omega \right|^2 + \left| \sum_{J,K} \chi^{(2)}_{YJK} \, E^J_\omega E^K_\omega \right|^2 \right) \tag{S3}$$

Here $\chi^{(2)}_{IJK}$ are the components of the susceptibility tensor in the microscope frame (X,Y,Z). One gets [7,10-14]:

$$I_{SHG}(\theta) = C\, I_\omega{}^2 \left[\left(\rho(\psi)\, cos^2(\theta - \varphi) + sin^2(\theta - \varphi) \right)^2 + \left(sin2(\theta - \varphi) \right)^2 \right] \tag{S4}$$

where I_ω is the intensity of the fundamental laser beam, C is a parameter merging geometrical and other parameters, and:

$$\rho = \chi_{XXX}/\chi_{XYY} = \rho_{fib} cos^2\psi + 3\, sin^2\psi \tag{S5}$$

The SHG signal thus depends on the incident polarization angle θ and on the orientation of the collagen fibrils in the microscope frame, that is the Euler angles to change from the microscope frame (X,Y,Z) to the collagen frame (x,y,z): the collagen angle φ in the imaging plane XY and the angle ψ out of the imaging plane. Since the collagen lamellae in the corneal stroma are composed of well-aligned fibrils, we consider here that all the collagen fibrils within the focal volume have the same orientation, in contrast to more complex approaches that introduce sub-micrometer orientation disorder [7,8]. Note that P-SHG is not sensitive to the polarity of collagen fibrils, like usual linear polarimetry, so that the φ and ψ angles are defined in smaller ranges than Euler angles: $\varphi, \psi \in]-90°, +90°]$.

1.2 Numerical processing of P-SHG

We use a FFT analysis to extract the collagen orientation φ from experimental P-SHG data in a fast and efficient way. Equation (4) is thus expressed as a function of its Fourier components:

$$I_{SHG}(\theta) \;=\; a_0(\psi) + \; a_2(\psi) cos[2(\theta - \varphi)] \;+\; a_4(\psi) cos[4(\theta - \varphi)] \tag{S6}$$

where:

$$a_0(\psi) = \mathrm{C}\; I_\omega{}^2\, cos^2(\psi) \begin{bmatrix} \frac{3\,cos^4\psi}{8} \chi^{(2)}_{xxx}{}^2 + cos^2\psi \left(\frac{5}{2} - \frac{9\,cos^2\psi}{4} \right) \chi^{(2)}_{xxx}\, \chi^{(2)}_{xyy} \\ + \left(5 - \frac{15\,cos^2\psi}{2} - \frac{27\,cos^4\psi}{8} \right) \chi^{(2)}_{xyy}{}^2 \end{bmatrix} \tag{S7}$$

$$a_2(\psi) = \; C\; I_\omega{}^2\, cos^2(\psi) \begin{bmatrix} \frac{cos^4\psi}{2}\, \chi^{(2)}_{xxx}{}^2 + 3\, cos^2\psi\, (1 - cos^2\psi) \chi^{(2)}_{xxx}\, \chi^{(2)}_{xyy} \\ + \left(4 - 9\, cos^2\psi + \frac{9\,cos^4\psi}{2} \right) \chi^{(2)}_{xyy}{}^2 \end{bmatrix} \tag{S8}$$

$$a_2(\psi) = C\ I_\omega{}^2\, cos^2(\psi)\left[\begin{matrix}\frac{cos^4\psi}{8}\,{\chi_{xxx}^{(2)}}^2 + cos^2\psi\left(\frac{1}{2}-\frac{3\,cos^2\psi}{4}\right)\chi_{xxx}^{(2)}\chi_{xyy}^{(2)} \\ +\left(\frac{9\,cos^2\psi}{8}-\frac{3\,cos^4\psi}{2}\right){\chi_{xyy}^{(2)}}^2\end{matrix}\right] \tag{S9}$$

FFT processing of P-SHG data then provides the coefficients α_0, α_2 and α_4 defined as:

$$I_{SHG}(\theta) = \alpha_0(\psi) + \alpha_2(\psi)e^{2i\theta} + \alpha_4(\psi)e^{4i\theta} + c.c. \tag{S10}$$

Our data-processing workflow thus provides 4 types of information in every pixel:

- The SHG signal averaged over all linear polarizations: $< SHG > = \langle I_{SHG}\rangle = \alpha_0$. This <SHG> intensity is similar to the usual SHG image acquired with circularly polarized excitation and same total acquisition duration.
- The in-plane orientation φ of the collagen fibrils, which corresponds to the first minimum of the polarimetric diagram $I_{SHG}(\theta)$ (Fig. 1.c). It is determined as the weighted average of the angles $\varphi_2 = -\frac{arg(\alpha_2)}{2}$ and $\varphi_4 = -\frac{arg(\alpha_4)\pm\pi}{4}$ [15]:

$$\varphi = \eta\varphi_2 + (1-\eta)\varphi_2 \quad \text{with} \quad \eta = \frac{|\alpha_2|^2}{|\alpha_2|^2+4|\alpha_4|^2} \tag{S11}$$

- A coefficient of determination R^2 that compares the experimental data and the curve obtained from the FFT parameters α_0, α_2 and α_4 in every voxel (Fig. S3). Indeed, the FFT analysis provide angles even if the experimental data do not match the theoretical equation (4) at all. Notably, the cylindrical symmetry does not apply in voxels encompassing crossing fibrils from sequential lamellae with different orientations. In this case, equation (4) is not valid anymore and higher order Fourier components are present in equation (6). We thus calculate:

$$R^2 = \max\left(0, 1-\frac{\Sigma_\theta[I_{SHG}(\theta)-I_{FFT}(\theta)]^2}{\Sigma_\theta[I_{SHG}(\theta)-\langle I_{SHG}(\theta)\rangle]^2}\right) \tag{S12}$$

where $$I_{FFT}(\theta) = \alpha_0 + \alpha_2 e^{2i\theta} + \alpha_4 e^{4i\theta} + c.c. \tag{S13}$$

Angles determined with $R^2<0.7$ are eliminated in the quantitative analyses and depicted as black pixels in the images. $R^2>0.7$ are used to code the brightness in orientation maps.

- The anisotropy parameter in the microscope frame, which corresponds to the square root of the ratio of the 2 minima: $\rho = \sqrt{\frac{I_{SHG}(\varphi)}{I_{SHG}(\varphi+\pi/2)}}$ (Fig. 1c) and is calculated as:

$$\rho = \sqrt{\frac{|\alpha_0|+2|\alpha_2|-2|\alpha_4|}{|\alpha_0|-2|\alpha_4|-2|\alpha_4|}} \tag{S14}$$

This parameter varies with ρ_{fib} and the out-of-plane angle ψ and can be used to estimate ψ. The transverse reconstructions nevertheless provide ψ in a more precise and easy way in this study.

This P-SHG processing workflow is implemented using Matlab (MathWorks Inc.).

References

1 Boyd, R. W. Nonlinear Optics. 2nd edn. (New York: Academic Press, 2003).

2 Roth, S. & Freund, I. Second harmonic generation in collagen. *Journal of Chemical Physics* **70**, 1637-1643 (1979).

3 Stoller, P. *et al.* Polarization-modulated second harmonic generation in collagen. *Biophysical Journal* **82**, 3330-3342 (2002).

4 Plotnikov, S. V. *et al.* Characterization of the myosin-based source for second-harmonic generation from muscle sarcomeres. *Biophysical Journal* **90**, 693–703 (2006).

5 Tiaho, F., Recher, G. & Rouède, D. Estimation of helical angle of myosin and collagen by second harmonic generation imaging microscopy *Optics Express* **15**, 12286-12295 (2007).

6 Tuer, A. E. *et al.* Nonlinear Optical Properties of Type I Collagen Fibers Studied by Polarization Dependent Second Harmonic Generation Microscopy. *Journal of Physical Chemistry B* **115**, 12759-12769, doi: 10.1021/Jp206308k (2011).

7 Duboisset, J. *et al.* Generic model of the molecular orientational distribution probed by polarization-resolved second-harmonic generation. *Physical Review A* **85**, 043829, doi: 10.1103/Physreva.85.043829 (2012).

8 Gusachenko, I. *et al.* Polarization-resolved second-harmonic microscopy in tendon upon mechanical stretching. *Biophysical Journal* **102**, 2220-2229 (2012).

9 Gusachenko, I. & Schanne-Klein, M.-C. Numerical simulation of polarization-resolved second harmonic microscopy in birefringent media. *Physical Review A* **88**, 053811 (2013).

10 Stoller, P. *et al.* Polarization-dependent optical second-harmonic imaging of a rat-tail tendon. *Journal of Biomedical Optics* **7**, 205-214 (2002).

11 Tuer, A. E. *et al.* Hierarchical Model of Fibrillar Collagen Organization for Interpreting the Second-Order Susceptibility Tensors in Biological Tissue. *Biophysical Journal* **103**, 2093-2105, doi: 10.1016/j.bpj.2012.10.019 (2012).

12 Psilodimitrakopoulos, S. *et al.* Effect of molecular organization on the image histograms of polarization SHG microscopy. *Biomedical Optics Express* **3**, 2681-2693 (2012).

13 Teulon, C. *et al.* Probing the 3D structure of cornea-like collagen liquid crystals with polarization-resolved SHG microscopy. *Optics Express* **24**, 16084-16098 (2016).

14 Rouede, D. *et al.* Determination of extracellular matrix collagen fibril architectures and pathological remodeling by polarization dependent second harmonic microscopy. *Scientific Reports* **7**, 12197, doi: 10.1038/s41598-017-12398-0 (2017).

15 Wasik, V. *et al.* Precision of polarization-resolved second harmonic generation microscopy limited by photon noise for samples with cylindrical symmetry. *Journal of the Optical Society of America A* **32**, 1437-1445, doi:10.1364/Josaa.32.001437 (2015).

2. Supplementary tables

Table S1: Physiological data, storage duration and thickness of the human corneas under study. * Storage duration in days between cornea collection and fixation. Storage conditions are described in the Materials and methods section.

** Thickness of the stroma measured after fixation using Optical Coherence Tomography (LC-OCT, Damae Medical, France). Uncertainty: ± 5 µm

N°	Donor sex	Donor age	Endothelial cell density (cell/mm^2)	Fixation date*	Stroma thickness (µm)**
1	F	71	1800	D27	600
2	F	59	2750	D22	490
3	F	56	not available	D24	490
4	F	63	2800	D22	410
5	F	88	1800	D26	470
6	H	83	1650	D26	460
7	H	71	2300	D22	330
8	H	57	2500	D20	380
9	H	61	2750	D18	630
10	H	81	1400	D33	520

3. Supplementary figures

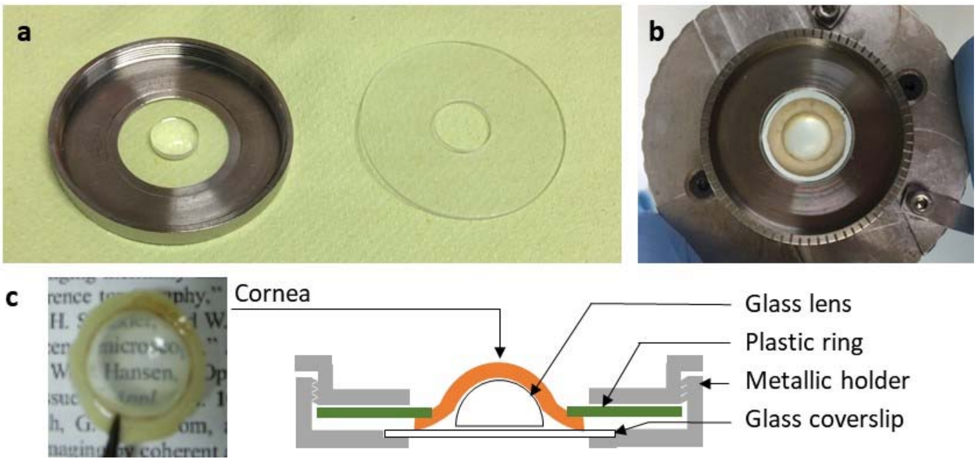

Figure S1. Cornea holder. (a) Picture of the parts of the cornea holder, showing the glass lens that maintains the corneal curvature and the plastic ring that clamps the sclera. (b) Picture of a mounted cornea. (c) Scheme of the cornea holder.

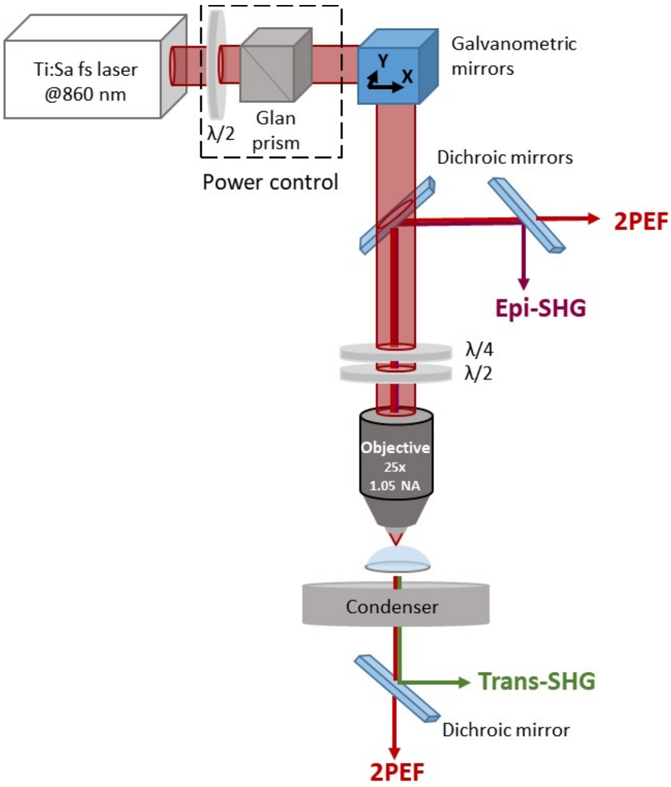

Figure S2. P-SHG setup

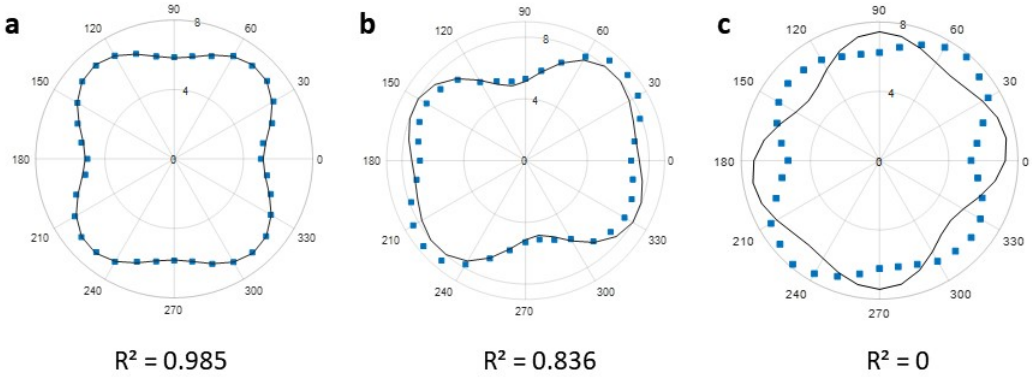

Figure S3. Calculation of the coefficient of determination R^2 for three different experimental P-SHG data. The polar plots display: (i) the experimental SHG signal as a function of the excitation polarization angle (blue solid dots) and (ii) the curve obtained by inserting the FFT parameters in the theoretical expression (Eqs. 3 or S4) (solid black line). a. Experimental data fit very well to the theory, so that R^2 is close to 1. b. Experimental data are slightly distorted and R^2 decreases. c. Experimental data do not follow Eq. 3 and R^2 is zero.

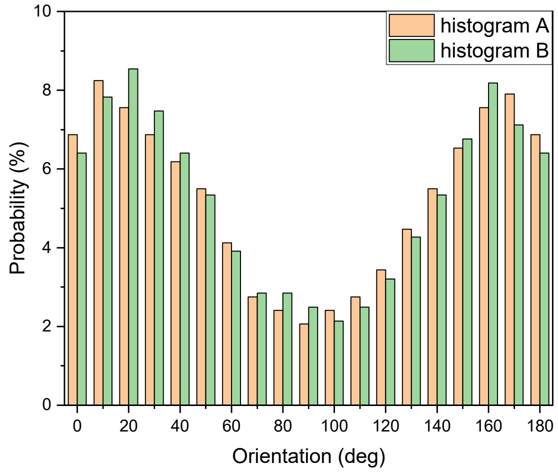

Figure S4: computation of the distance of 2 distributions A and B. The distance is calculated as the summation of the absolute difference between the distribution values at each angle: $D(A,\, B) = \sum_{\varphi} |h_A(\varphi) - h_B(\varphi)|$. It is then normalized to the maximum distance between two normalized distributions, *ie* divided by 2.

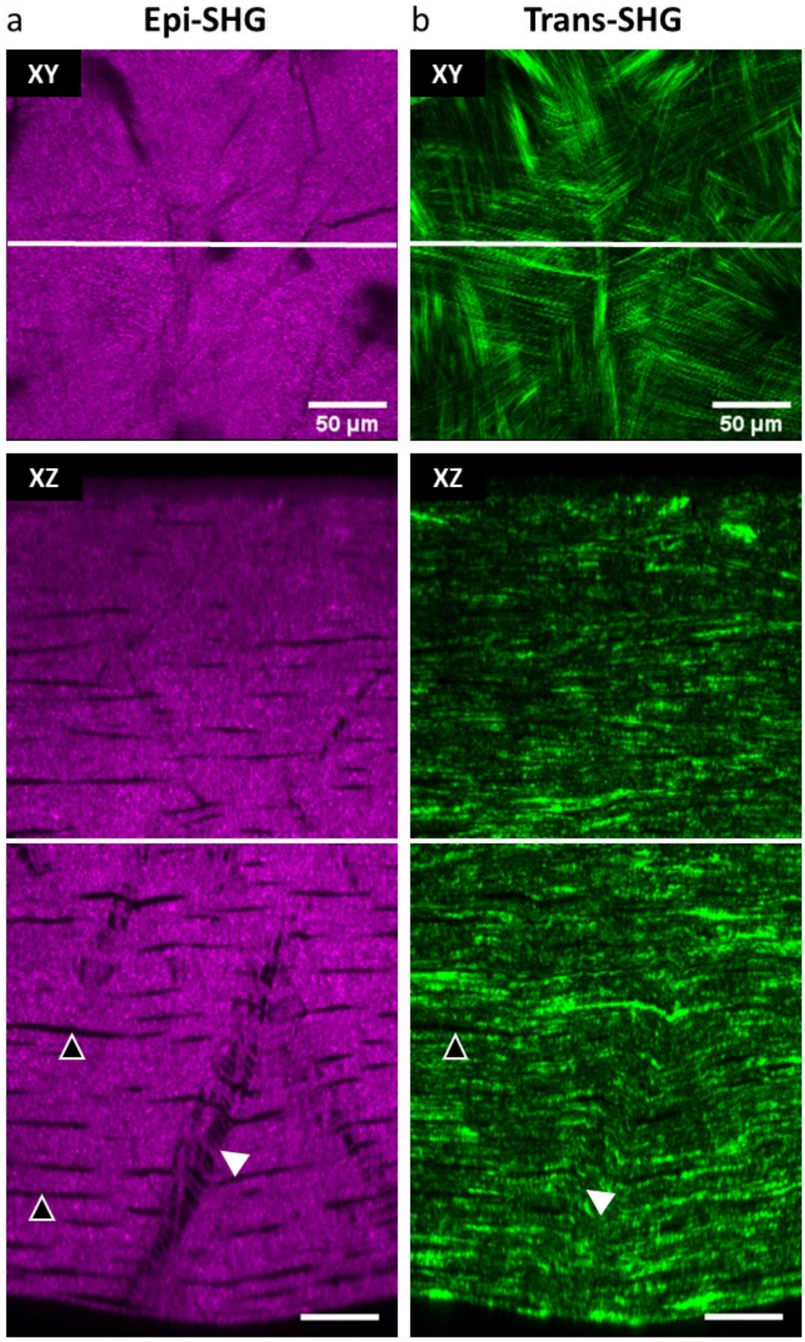

Figure S5. *En face XY* <SHG> images and transverse XZ reconstruction of a human cornea (Cornea n°10 in Supplementary Table S1) using (a) epi-detection and (b) trans-detection. The white lines on the XY images indicate the position of the XZ reconstruction and vice-versa. White arrow heads show stromal striae and black arrow heads show keratocytes.

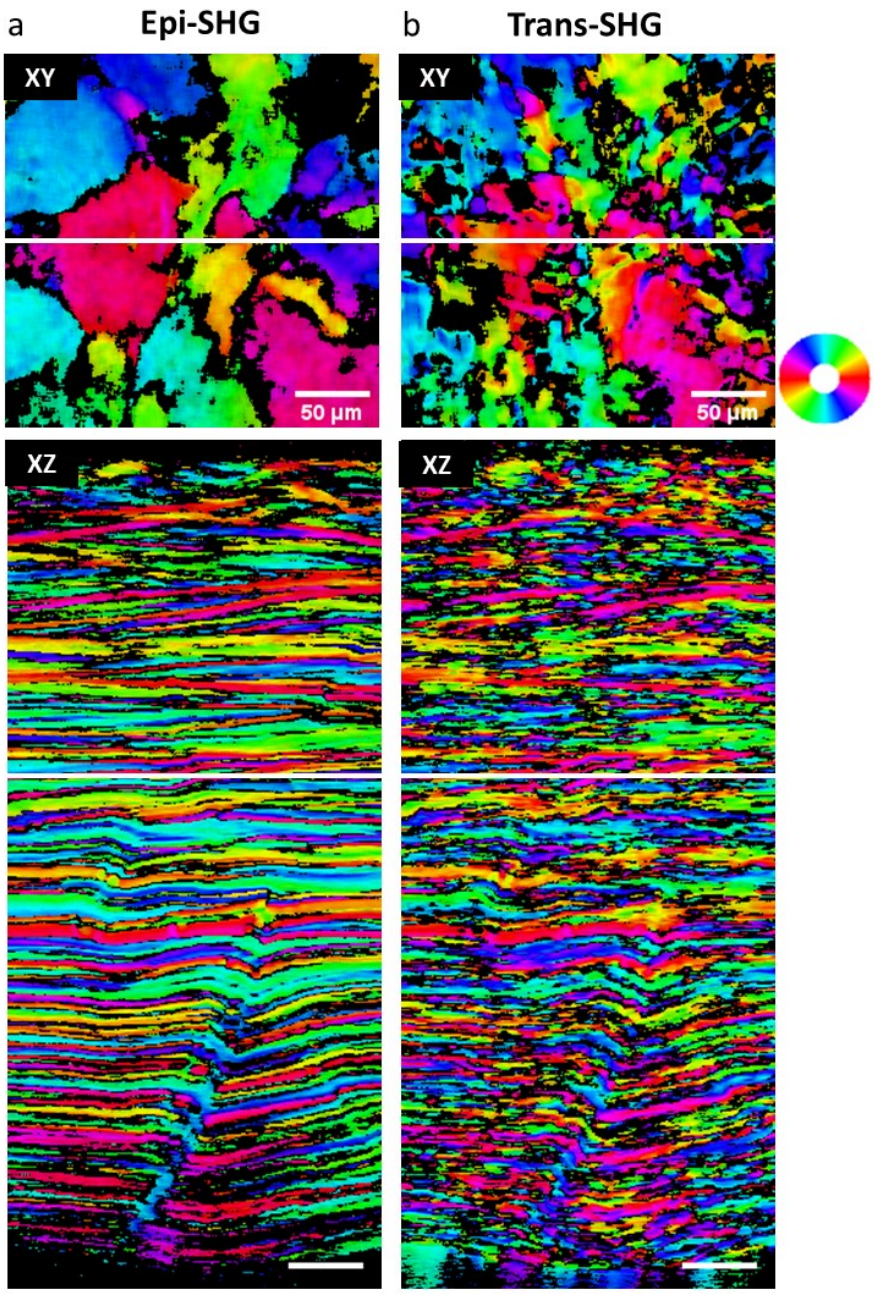

Figure S6. *En face XY* orientation map and transverse XZ reconstruction processed from P-SHG imaging of the same human cornea as in figure S3 (Cornea n°10 in Supplementary Table S1) using (a) epi-detection and (b) trans-detection. The white lines on the XY images indicate the position of the XZ reconstruction and vice-versa. HSV look-up-table is used: H codes the orientations, as indicated on the color wheel; S=1 and V corresponds to R^2. Voxels with R^2<0.7 are filtered out and depicted in black.

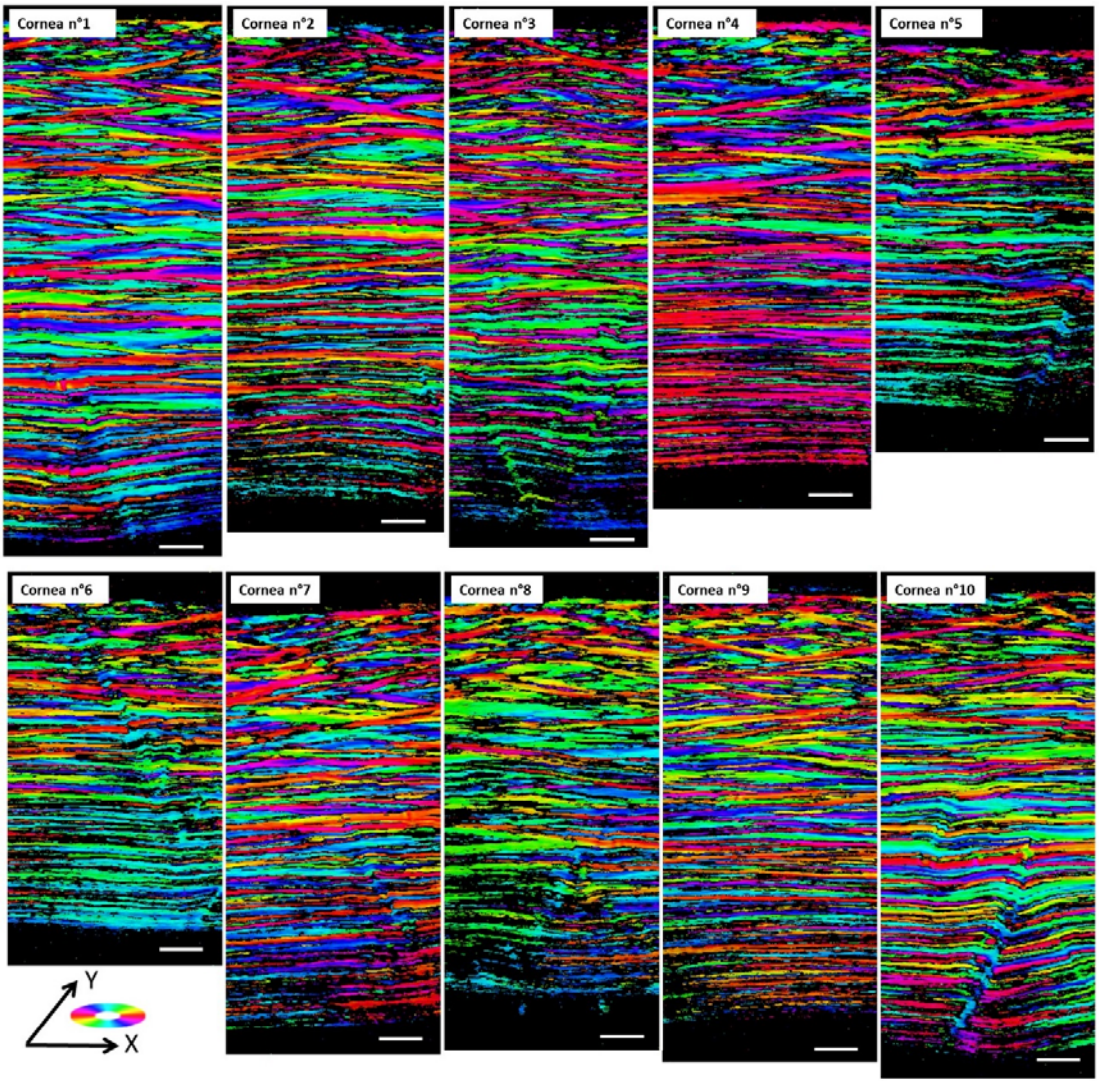

Figure S7. Transverse reconstructions of collagen orientation processed from *en face* P-SHG imaging of 10 human corneas using epi-detection. HSV look-up-table is used: H codes the orientations, as indicated on the color wheel; S=1 and V corresponds to R^2. Voxels with $R^2<0.7$ are filtered out and depicted in black. Scale bar: 50 µm.

4. Supplementary Movies

Movie 1. P-SHG imaging of a human cornea (Cornea n°1 in table S1) over its full thickness. Left: *en face* Z-stacks from the epithelium to the endothelium showing (top) the epi-detected <SHG> image and (bottom) the collagen in-plane orientation. Right: transverse reconstruction showing (top) the epi-detected <SHG> image and (bottom) the collagen in-plane orientation. The yellow line on the transverse reconstructions indicates the depth Z of the *en face* images.

Movie 2. Protocol to obtain reliable orientation distribution of collagen in the posterior stroma: (i) acquisition of 2 P-SHG image stacks in the same cornea oriented with the naso-temporal axis along the *X* (left) and *Y* (right) directions; (ii) calculation of the registration angle (close to 90°) of the 2 orientation distributions by minimizing their distance (ii) rotation by this registration angle of the orientation distribution extracted from the second P-SHG stack; (iii) averaging of this rotated distribution with the distributions extracted from the first P-SHG stack.
